# The Newborn's Reaction to Light as the Determinant of the Brain's Activation at Human Birth

**DOI:** 10.3389/fnint.2022.933426

**Published:** 2022-09-02

**Authors:** Daniela Polese, Maria Letizia Riccio, Marcella Fagioli, Alessandro Mazzetta, Francesca Fagioli, Pasquale Parisi, Massimo Fagioli

**Affiliations:** ^1^PhD Program on Sensorineural Plasticity, Department of Neuroscience, Mental Health and Sensory Organs NESMOS, Sant'Andrea Hospital, Sapienza University of Rome, Rome, Italy; ^2^Department of Medical Biotechnologies, University of Siena, Siena, Italy; ^3^Department of Mental Health, National Health System ASL Rome 1, Rome, Italy; ^4^PhD Program on Neuroscience, Department of Systems Medicine, Tor Vergata University, Rome, Italy; ^5^Chair of Pediatrics, Department of Neuroscience, Mental Health and Sensory Organs NESMOS, Sant'Andrea Hospital, Sapienza University of Rome, Rome, Italy; ^6^Via Roma Libera, Rome, Italy

**Keywords:** human birth, light, retina, first breath, newborn, intrinsically photosensitive retinal ganglion cells (ipRGCs), melanopsin, IEGs

## Abstract

Developmental neuroscience research has not yet fully unveiled the dynamics involved in human birth. The trigger of the first breath, often assumed to be the marker of human life, has not been characterized nor has the process entailing brain modification and activation at birth been clarified yet. To date, few researchers only have investigated the impact of the extrauterine environment, with its strong stimuli, on birth. This ‘hypothesis and theory' article assumes the role of a specific stimulus activating the central nervous system (CNS) at human birth. This stimulus must have specific features though, such as novelty, efficacy, ubiquity, and immediacy. We propose light as a robust candidate for the CNS activation *via* the retina. Available data on fetal and neonatal neurodevelopment, in particular with reference to retinal light-responsive pathways, will be examined together with the GABA functional switch, and the subplate disappearance, which, at an experimental level, differentiate the neonatal brain from the fetal brain. In this study, we assume how a very rapid activation of retinal photoreceptors at birth initiates a sudden brain shift from the prenatal pattern of functions to the neonatal setup. Our assumption implies the presence of a photoreceptor capable of capturing and transducing light/photon stimulus, transforming it into an effective signal for the activation of new brain functions at birth. Opsin photoreception or, more specifically, melanopsin-dependent photoreception, which is provided by intrinsically photosensitive retinal ganglion cells (ipRGCs), is considered as a valid candidate. Although what is assumed herein cannot be verified in humans based on knowledge available so far, proposing an important and novel function can trigger a broad range of diversified research in different domains, from neurophysiology to neurology and psychiatry.

## Introduction

Conventionally, birth begins with the first breath. This tenet is widespread and has major implications in several fields, including ethics, law, and end-of-life care. The terms and concepts revolving around birth and the beginning of human life are often flawed by multiple competing hypotheses, which tend to be based on humanistic and/or cultural beliefs rather than scientific merits (Dupont-Thibodeau and Janvier, 2016; Polese et al., 2021). Observational studies define birth's time *0* as the moment when the newborn's thorax and pelvis pass through the birth canal (Rousseau et al., 2014). In this study, we take into account the existence of a hiatus between intrauterine and extrauterine condition, and only use the terms ‘human life and human birth,' to define the newborn's extrauterine existence, given new physiological conditions (Hillman et al., 2012; Morton and Brodsky, 2016; Schwindt et al., 2018; Schmidt Mellado et al., 2022).

Every stage of childbirth, including labor, expulsion and coming into contact with a different environment, is stressful (Lagercrantz and Bistoletti, 1977; Lagercrantz and Slotkin, 1986). Between birth and the first breath, there is some time, about 20 s, during which the newborn does not breathe but physiological events occur, including lung fluid clearance. We assume these phenomena might represent the newborn's reaction to the new, stressful extrauterine environment, as opposed to the previous intrauterine condition, started by the central nervous system (CNS; Polese et al., 2021). Mechanisms are activated in order to initiate and sustain a number of functions that are essential for survival (Morton and Brodsky, 2016; Stroud et al., 2020; Shi et al., 2021). This activation characterizes the newborn's physiology as soon as he or she is exposed to the extrauterine environment before the first breath happens. Time before breathing can physiologically last up to 60 s (WHO, 2012); however, the triggers of physiological events and what occurs at the CNS level at this time have not been discovered yet (Polese et al., 2021).

During gestation, the structural and functional development of the CNS depends on a complex array of coordinated ontogenetic events, such as neuronal proliferation and migration, synaptogenesis, apoptosis and circuit formation (for review, see de Graaf-Peters and Hadders-Algra, 2006; Kostović et al., 2021). The neuroanatomical and functional organization as well as the transcriptional profiling of the human fetal brain show unique features compared to other mammals and primates (Kostović and Rakic, 1990; Ulfig et al., 2000; Miller et al., 2014; Molnár et al., 2019). By mid-gestation, the architecture of the human fetal forebrain is characterized by functionally relevant transient structures that disappear at term age, including the subplate zone, a transient cortical compartment that is extraordinarily expanded in humans (Kostović and Rakic, 1990; Ulfig et al., 2000; Kostović and Judaš, 2010). In humans and other primates, in the second half of pregnancy, developing neural circuits show patterns of endogenously generated electrical activity that contribute to the development and wiring of neuronal networks (for review, see Ben-Ari et al., 2007; Blankenship and Feller, 2010). These patterns of spontaneous activity differ from the patterns of sensory-driven activity that characterize neonatal neuronal networks, and are sustained by cellular mechanisms that are typical of developing circuits, such as the depolarizing action of gamma-aminobutyric acid (GABA), extra-synaptic transmission, gap junction coupling and the presence of pacemaker-like neurons (Ben-Ari et al., 1989, 2007; Spitzer, 2006; Crépel et al., 2007; Blankenship and Feller, 2010; Colonnese and Khazipov, 2012; Arroyo and Feller, 2016).

This spontaneous network activity has been described to be robust as it is homeostatically regulated and actively maintained by strong compensatory mechanisms (Blankenship and Feller, 2010). The activity has been observed in many developing neural circuits. For instance, the immature retina exhibits highly correlated bursts of action potentials (retinal waves) that are transmitted to the entire developing visual system, thereby providing a robust source of activity before the onset of visual experience (Khazipov and Luhmann, 2006; Huberman et al., 2008; Colonnese et al., 2010; Maccione et al., 2014; Arroyo and Feller, 2016; Ge et al., 2021). Likewise, spontaneous bursts of activity *in utero*, produced by central pattern generators (CPGs) in the spinal cord, cause myoclonic jerks in fetal muscles, resulting in spontaneous sensory input that is carried to the somatosensory cortex (Khazipov et al., 2004; Colonnese and Khazipov, 2012).

Studies on animal models have shown that the emergence of immature, spontaneous neuronal networks follows a triphasic developmental sequence. Initially, at an early embryonic stage, voltage-gated, non-synaptic calcium currents are generated, followed by large calcium plateaux in small neuronal populations connected by gap junctions. Subsequently, first spontaneous synapse-driven patterns appear like retinal waves in the visual system and the so-called ‘giant depolarizing potentials' (GDPs) in the immature hippocampal neurones, which synchronize the entire network (Ben-Ari et al., 1989; Crépel et al., 2007; for review see Ben-Ari and Spitzer, 2010; Ben-Ari, 2014). Moreover, these studies have demonstrated that the GABA excitatory/inhibitory developmental sequence has a key role in the complex occurence of events associated with the development of these patterns (Crépel et al., 2007; for reviews, see Ben-Ari et al., 2007; Ben-Ari and Spitzer, 2010; Ben-Ari, 2014). Although the timing when these patterns develop varies in brain structures and in different animal species, their developmental sequences are comparable. It is worth noticing how these sequences appear at different stages in different species; still, they are similar in postnatal rodents and *in utero* primates (Ben-Ari et al., 1989, 2007, 2012; Khazipov et al., 2001; Cherubini and Ben-Ari, 2011).

In the retina, as in other developing circuits, spontaneous neuronal activity disappears quickly after birth, in parallel with the maturation of functional GABAergic inhibition (Ben-Ari et al., 1989; Sernagor et al., 2003; Blankenship and Feller, 2010; Colonnese and Khazipov, 2010; Colonnese et al., 2010; Maccione et al., 2014; Romagnoni et al., 2020). In humans and other primates, spontaneous retinal waves occur at a prenatal stage only (considering neonates at term), whereas in other species, such as mice and ferrets, which are more immature at birth, they occur both prenatally and postnatally, disappearing around the time of eye opening (Huberman et al., 2008; Colonnese et al., 2010). In mice and ferrets, retinal waves and sensory-driven retinal activity coexist after birth for a few days around eye-opening, after which light-evoked responses dominate (Colonnese et al., 2010; Maccione et al., 2014; Tiriac et al., 2018). In humans, visually driven activity begins at birth. In particular, in premature infants, early visual responses are strongly amplified by the immature thalamocortical network and they mature in parallel with the downregulation of spontaneous waves (Colonnese et al., 2010). Moreover, a switch in sensory processing has been identified. Interestingly enough, this switch prepares developing neocortex for vision and has been causally associated with the emergence of a continuous cortical activity, dependent on the neuromodulatory ascending arousal system (Colonnese et al., 2010). However, the factors that determine this neonatal switch have not been clarified yet (Sernagor et al., 2003; Colonnese et al., 2010; Romagnoni et al., 2020).

In the human fetus, electroencephalography (EEG), magnetoencephalography (MEG) and functional magnetic resonance imaging (fMRI) studies have shown spontaneous neural activity, which occurs naturally, without any direct stimulation (Anderson and Thomason, 2013). On the other hand, one of the features of postnatal brain development is its dependence on sensory experience (Wiesel and Hubel, 1963). At human birth, changes in the CNS physiology occur together with the well-known physiological changes that characterize the neonatal condition, meaning cardiovascular and pulmonary changes (Morton and Brodsky, 2016; Stroud et al., 2020; Hoffiz et al., 2021).

In the last few weeks of gestation, the CNS develops dramatically; transient fetal brain structures resolve (Kostović and Rakic, 1990; de Graaf-Peters and Hadders-Algra, 2006); and neurotransmitter systems, particularly the GABAergic and the glutamatergic systems, undergo marked changes (Ben-Ari et al., 2007; Romagnoni et al., 2020). These developmental changes are functional for the child's interaction with the extrauterine environment (Gatti et al., 2012; Basu et al., 2021).

At birth, abrupt and intense sensory stimulation by the extrauterine environment can play an essential role in driving the subsequent development of full-brain functions. In particular, the extraordinary sensory input from the new environment can be the triggering event that activates the whole brain at birth, determining the first breath (Cohen and Katz-Salamon, 2005; Polese et al., 2021). Recently, a brainstem signaling system, which is upregulated at birth to support breathing, has been reported in experimental models (Shi et al., 2021). Even more intriguingly, in humans, a pontine reflex blocking center, which prevents respiratory motion in the fetus, has been shown to reverse quickly to a respiratory-facilitating function immediately at birth, giving rise to the first inspiratory action (Ottaviani, 2014; Lavezzi et al., 2016).

Although data seem controversial, comparative studies on the human fetal brain and the neonatal brain confirm that activity patterns differ dramatically (Giménez et al., 2008; Doria et al., 2010; Viola et al., 2011; Cao et al., 2017; Counsell et al., 2019). Immature brain activity in preterm newborns, once these are exposed to the external environment, changes and displays EEG features similar to full-term, mature brains (Vanhatalo et al., 2005; Schwindt et al., 2018). Likewise, in MRI studies, the immature brain of preterm babies shows acceleration in maturation and becomes similar to the brain of full-term newborns of the same age (Giménez et al., 2008; Doria et al., 2010; Viola et al., 2011). Indeed, resting-state fMRI data show how the brain of preterm newborns differs from that of a healthy fetus of the same age, with stronger connectivity in sensory input and stress-related areas after contact with the extrauterine environment (De Asis-Cruz et al., 2020).

Although stimuli from the external environment (*Umwelt*) are multifactorial (André et al., 2018), we consider that one single-specific stimulus is likely to work as a trigger for the brain shifting from a fetal pattern of functions to a distinctive neonatal setup so as to determine the first breath (Polese et al., 2021). Given the important role of sensory input in neurodevelopment, new research has focused on the extrauterine environment's dramatic impact at birth (Tsuneishi and Casaer, 2000; Viola et al., 2011; Schwindt et al., 2018; De Asis-Cruz et al., 2020). Extrauterine stimulation is a fundamental factor when considering brain maturation. After exposure to the extrauterine environment, the premature newborn's neurodevelopment is accelerated, in particular in relation to visual and tactile response (Schmidt Mellado et al., 2022). At birth, exposure to this new hyper-stimulating environment happens abruptly and brain reaction is needed in a few seconds. If this physiological reaction does not occur within 60 s, cardiopulmonary resuscitation is required. This reaction would fit with the time needed for physiological changes occurring before the first breath (LoMauro and Aliverti, 2016; Morton and Brodsky, 2016).

This single new stimulus, responsible for starting brain activity at human birth, should be efficient, meaning, it has to ensure birth, for the survival of the human species, it should always be available in the environment and should work fast, in only a few seconds (~20 s), between exit from the uterus and the newborn's first breath and crying.

At birth, the dramatic functional transformation occurring at the level of the CNS may be determined by the brain's reaction to external stimuli. For instance, the capability to react to external stimuli characterizes humans as it is essential for viability of both preterm and full-term newborns (Gatti et al., 2012). Moreover, newborns can interact with other human beings as early as birth (Brazelton and Nugent, 2011; Cusack et al., 2016; Fagioli, 2019).

The aim of this ‘hypothesis and theory' article is to provide some perspectives, to be confirmed at an experimental level, on a likely stimulus that is involved in the human CNS primal response to the new environment at birth. In particular, we shall consider how light can be directly involved in this response.

## Brain Activation and the First Breath

In the uterus, stimuli from the extrauterine environment are not directly perceived by the fetus. Every stimulus is buffered by amniotic fluid, which is the only thing the fetus has contact with. Any disturbance to or fluctuation in homeostasis, including mechanical stimuli or vibrations due to an accident or noise, is mediated by amniotic fluid to maintain intrauterine homeostasis. Minor, buffered disturbance can work as endogenous stimulation without disrupting fetal homeostasis. As mentioned before, only spontaneous fetal jerks only can determine somatosensory stimuli (Khazipov et al., 2004; Colonnese and Khazipov, 2012). This provides physiological protection to fetal development, for the survival of human species. On the contrary, at birth, the extrauterine environment offers no mediation; the newborn is abruptly exposed to the external world where multiple sensory inputs, from direct and powerful exogenous stimuli, begin to modulate neonatal cortical networks (Lagercrantz and Slotkin, 1986; André et al., 2018; Fagioli, 2019).

**Figure 1:**
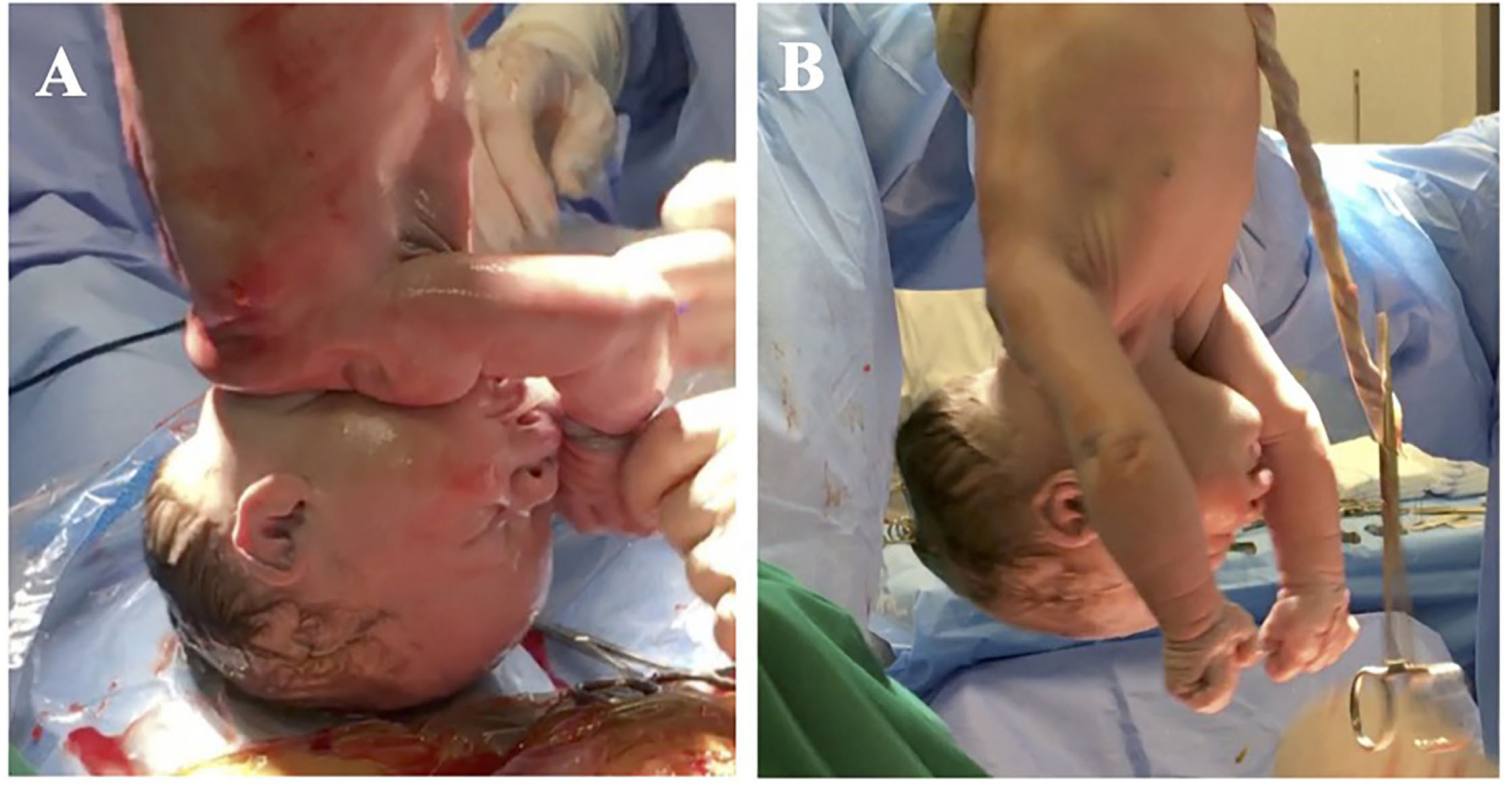
The newborn immediately after birth. **(A)** Shows a baby 4 s after birth, before the first breath occurs. Lungs are collapsed so that a large cavity is visible on the thorax. Muscles are flaccid and hang down. **(B)** Shows a baby 12 s after birth, after the first breath, before crying. The cavity on the baby's thorax is no longer visible and the muscles are visibly tonic. The color of the skin has changed, due to oxygenation.

The beginning of respiratory activity has been associated with several mechanisms such as sub-atmospheric intrathoracic pressure, the release of fetal cortisol and adrenaline, which mediate the activation of sodium channels, the release of arginine, vasopressin and copeptin, the secretion of surfactant by alveolar cells and, in the case of natural childbirth, the release of maternal oxytocin, uterine contraction during labor, and the passage through the birth canal (The first breath, 1959; Faxelius et al., 1983; Uvnäs-Moberg et al., 2019; for review, see te Pas et al., 2008; Evers and Wellmann, 2016; LoMauro and Aliverti, 2016; Ben-Ari, 2018). However, at birth, lung fluid clearance occurs in a few seconds (~20 s) and coincides with the first breath (van Vonderen et al., 2014; LoMauro and Aliverti, 2016), while stress hormone release, such as catecholamines, begins before birth (Lagercrantz and Bistoletti, 1977; Lagercrantz and Slotkin, 1986), also the activation of sodium channels is expected to take some time (van Vonderen et al., 2014). Before the first breath, the newborn does not show any motory activity and he or she is physiologically flaccid (Figure 1). The beginning of respiratory activity does not even appear to be associated with chemoreceptor responses, as chemoreceptors are not fully developed at birth, becoming functional from a few days to a few weeks after birth (Cohen and Katz-Salamon, 2005).

Moreover, since lung fluid clearance and breathing occur during both delivery with labor and in caesarian delivery (C-section), there must be a common mechanism in place that is at the basis of the beginning of breathing. In order for the first breath to take place, the diaphragm should contract and flatten and the chest cavity enlarge (Figure 1). This contraction creates a vacuum that pulls air into the lungs. Upon exhalation, the diaphragm relaxes and returns to its domelike shape, and the air is forced out of the lungs. Thus, it can be assumed that the strength that is exerted by the diaphragm's contraction determines fluid shift, lung fluid clearance, and the first breath (LoMauro and Aliverti, 2016; Polese et al., 2021). At birth, in the absence of functional chemoreceptors, abrupt and intense brain stimulation by the extrauterine environment, namely, a ‘neurogenic respiratory drive', may trigger and sustain the diaphragm contraction and the first breathing effort (Cohen and Katz-Salamon, 2005; Polese et al., 2021).

Moreover, central respiratory networks in the brainstem quickly lose their inhibitory function and begin to generate continuous, rather than intermittent, breathing movements (van Vonderen et al., 2014; LoMauro and Aliverti, 2016). Evidence in human studies show that, in the near-term fetus, the pontine parabrachial/Kölliker-Fuse (KF) complex accounts for the strong inhibitory action of chemoreflexogenesis. At birth, the function of the KF nucleus changes abruptly, reversing to a respiration-facilitating function. Under the stimulation of the KF nucleus, the pontine facial/parafacial complex starts working at birth, triggering the medullary pre-Bötzinger nucleus which is the actual activator of the diaphragm contraction and of the first respiratory action (Ottaviani, 2014; Lavezzi et al., 2016). However, what triggers this sudden switch from respiratory suppression to stimulation at birth has not been determined yet.

## Environmental Stressors at Human Birth and the Possible Role of the Retina

The newborn comes from a homeostatic condition, which is characterized by warmth, darkness, quiet, and protection. On the contrary, the extrauterine world, even when it is strictly controlled and kept under optimal conditions, is characterized by handling, cold, noise, and light. As mentioned before, due to the strain of the childbirth process, the fetus releases an extraordinary amount of stress hormones, at even higher levels than mothers during labor or adults suffering from myocardial infarction (Lagercrantz and Bistoletti, 1977; Bistoletti et al., 1983; Lagercrantz and Slotkin, 1986; Greenough et al., 1987). Birth is the life event when the most dramatic physiologic changes occur so that a massive adaptation is needed at this stage (Hillman et al., 2012; André et al., 2018; Doherty et al., 2021). Several hormones causing analgesia are released and a subsequent reduction in nociceptive response by the newborn has been observed immediately after labor. Delivery is a stressful and potentially painful event for newborns (Lagercrantz and Bistoletti, 1977; Bistoletti et al., 1983; Hägnevik et al., 1984; Bergqvist et al., 2009; Mazzuca et al., 2011). Oxytocin serves multiple functions including analgesia and anti-inflammatory activity (for review, see Ben-Ari, 2018). It has a fundamental role at birth, as it causes pain relief *via* reduction in depolarizing activity of GABA on nociceptive neurons, lasting few hours during and after normal delivery (Bergqvist et al., 2009; Mazzuca et al., 2011). On the other hand, elective C-section is associated with higher nociceptive response values a few hours after birth (Bergqvist et al., 2009; Mazzuca et al., 2011; Kasser et al., 2019), with lower protection against pain. Although C-section without labor is less stressful, it still entails the release of fetal catecholamines, though at a lower level (Hägnevik et al., 1984). Moreover, unlike in rats, in humans, the oxytocin-mediated newborn analgesia is always present, as it correlates with the release of fetal oxytocin (Mazzuca et al., 2011). Hence, regardless of the type of delivery, all childbirth processes appear to cause great strain, with the same need to adapt to the new environment.

The release of catecholamines is crucial for the newborn's survival. It has been reported that, even with intense and prolonged hypoxia during labor, extraordinarily high levels of catecholamines make the body's ‘fight' for survival possible, enabling the fetus to withstand low oxygen conditions before and during delivery. Catecholamines increase fetal heart rate, enhance the newborn's ability to function effectively when it is first separated from the mother, and allow for the absorption of lung fluid and the release of adequate surfactant in the hours before birth, in order to determine a respiratory competence (Lagercrantz and Bistoletti, 1977; Lagercrantz and Slotkin, 1986; Greenough et al., 1987). Even if catecholamines prepare the fetus to be born and have a crucial role in birth, they are secreted before delivery and could not be the actual trigger of brain activation, of ‘neurogenic drive' and the first breath. It has been reported that fluid clearance occurs almost exclusively (>95%) during inspiration after birth (van Vonderen et al., 2014; Hooper et al., 2015). The intense brain activation, which happens at birth, is enough to begin respiration. In the days or weeks that follow, this neurogenic drive weakens and the drive from chemoreceptors becomes crucial for generating and maintaining a normal breathing rhythm (Forster et al., 2000; Cohen and Katz-Salamon, 2005).

Given the changes occurring from the prenatal brain to the postnatal brain, we aimed at characterizing the very early stimuli that trigger these changes. Among several diversified candidates, only one single specific activating agent only is characterized by the above-mentioned qualities, meaning novelty, impact, immediacy, and ubiquity. Moreover, we can speculate that this stimulus can reach the brain directly, without any intermediate transfer of information (Polese et al., 2021). Light, which hits the retina, has all these features. Despite its peripheral location, the retina is part of the CNS and is the sole CNS portion that is ‘approachable' (for review, see Dowling, 1987). In humans, as in all mammals, the retina is the only structure that catches and rapidly photo-transduces light, transforming photon energy into electrical signals and conveying this information to the rest of the brain *via* the optic nerve (Hattar et al., 2003).

Like the rest of the brain, the retina has a layered structure and comprises a great diversity of neuronal cell types. Humans and simian primates possess a unique retinal central architecture with an unusual spatial distribution of neurons, namely, the fovea, which enables high acuity vision (for review, see Provis and Hendrickson, 2008). In all mammals, the basic retinal architecture includes an array of visual photoreceptors, i.e., rods and cones (the input elements), which transduce absorbed light into electrical activity, as well as an array of ganglion cells that encode this activity as a train of action potentials carried along the axonal fibers of the optic nerve (for review, see Masland, 2012). Retinal photoreceptors express opsin-based photopigments with different spectrum maximum sensitivity (λ_max_) but overlapping spectral responses. In humans, the visible light spectrum has a wavelength ranging from ~400 to 700 nm (but see: Sliney, 2016). Unlike most mammals, humans and some simian primates have three different cones, expressing opsins with peak sensitivity to short (~430 nm), medium (~530 nm), or long (~560 nm) wavelengths (Jacobs, 2008). Cone photoreceptors are involved in photopic (bright light) color vision and acuity (Stockman et al., 2000). Rod photoreceptors, which express rhodopsin with a peak sensitivity of around 500 nm wavelength, are specialized in scotopic (dim light) vision, providing low-resolution but high sensitivity (Schnapf et al., 1988).

Remarkably, the photochemical reaction of retinal opsin-based photopigments happens with quantum efficiency in a few femtoseconds and is one of the most rapid and efficient processes existing in nature (Schoenlein et al., 1991; Polli et al., 2010; Pugh, 2018).

Retinal ganglion cells (RGCs) are the only retinal output neurons. They receive processed rod/cone input through the retinal circuitry and send this visual information to image-forming brain targets, such as the dorsal lateral geniculate nucleus (dLGN) and superior colliculus (SC), and finally to the primary visual cortex (Masland, 2012).

However, sight is not the only function of the retina. In addition to standard visual functions, characterized by elevated spatial and temporal contrast acuity, the retina encodes environmental irradiance (the overall intensity of illumination) to regulate several aspects of our physiology, such as circadian rhythms, pupillary light reflex (PLR), hormone synthesis, and behavioral responses (Van Gelder, 2008). These light responses are maintained in blind animal models (Foster et al., 1991; Freedman et al., 1999) and in blind human subjects lacking functional rods and cones (Czeisler et al., 1995), and are, therefore, referred to as non-image-forming or ‘non-visual.' Thus, the mammalian eye contains two different photoreceptive systems, i.e. the standard visual system for conscious perception of images and a not-rod/not-cone-mediated, ‘non-visual' system for functions that occur beyond conscious perception. The mammalian retina has always been thought to contain one single photoreceptive system, in the early 2000's, a second photoreceptive system has been characterized based on evidence showing the existence of a third type of retinal photoreceptors (Provencio et al., 2000; Berson et al., 2002; Hattar et al., 2002). Also, it has been shown that only this second, non-visual photoreceptive system is mature at birth, ‘[...] second sight comes first [...].' (Sernagor, 2005). Thus, the discovery of this new retinal pathway is to be considered remarkable within the context of our research.

## IPRGCS' Non-Visual Pathway

This new class of photoreceptors mostly provides non-visual light responses that are essential to our physiology and health, including our internal clock's sinchronization to the solar day (circadian photoentrainment; Provencio et al., 2000; Berson et al., 2002; Hattar et al., 2002). These novel photoreceptors have been detected in an unlikely place in the retina. The standard visual photoreceptors, i.e., rods and cones, are located on the outer nuclear layer of the retina, farthest from the pupil. At the opposite side, on the inner retinal layer, closest to the incoming light, there are the retinal ganglion cells (RGCs), which are the sole output neurons of the retina whose axons exit the eye and project to retinorecipient brain nuclei (Masland, 2012). Surprisingly enough, apart from acting as conventional RGCs, a very small number of RGCs also express novel opsin-based photopigment melanopsin that makes them intrinsically photosensitive (ipRGCs; Provencio et al., 2000; Berson et al., 2002; Hattar et al., 2002).

As shown in Table 1, where the different features of the human visual and non-visual photoreceptive systems have been highlighted, melanopsin photoreception is mostly sensitive to short-wavelength blue light, with a peak close to 480 nm, which differs significantly (≥ 20 nm) from the best wavelengths stimulating rod and cones opsins (Berson et al., 2002; Hattar et al., 2003; Cajochen et al., 2005; Dacey et al., 2005; Zaidi et al., 2007). However, melanopsin-expressing cells have been shown to respond to an unusually broad interval of the visible spectrum thanks to conformational changes in melanopsin (Do, 2019). An unusual physiologic feature of ipRGCs consists in an extraordinarily prolonged response to light, which lasts dozens of times longer than rod/cone responses and persists long after light stimulus has disappeared (Berson et al., 2002; Do, 2019). Like rods, ipRGCs have been shown to signal single-photon absorption, while melanopsin has been shown to have quantum efficiency comparable to (Do et al., 2009), or even greater (Rinaldi et al., 2014) than that of rhodopsin and cone opsins. However, ipRGCs have been described to be less sensitive than rods and cones, and to operate at higher light irradiances (Berson et al., 2002; Dacey et al., 2005; Mure et al., 2019), which may be due to their lower per-cell photopigment density that reduces the possibility of catching photons (Do et al., 2009; Rinaldi et al., 2014; Table 1). However, evidence suggests that ipRGC pigment expression, and thus relevant cellular density, is not constant but adjusts itself (Hannibal et al., 2005; Sekaran et al., 2005; Tu et al., 2005). In particular, ipRGC melanopsin density has been shown to increase in darkness and decrease in constant light (Hannibal et al., 2005). Also, regardless of melanopsin concentration, ipRGCs' light sensitivity or response amplitude has been shown to be higher in the dark-adapted retina (Zhao et al., 2014).

**Table 1:** Human visual and “non-visual” photoreceptive systems.

	**Visual pathway**		**‘Non-visual' pathway**
Photoreceptor cells	Rods	Cones	ipRGCs
Location	Outer retina	Outer retina	Inner retina
Peak spectral absorbance (λ_max_)	~500 nm	~430 nm ~530 nm ~560 nm	~480 nm
Photopigment density/cell	High	High	Low (1/10,000 rhodopsin density)
Total number of cells/retina	~1,2 × 10^8^	~6 × 10^6^	4-7 × 10^3^
Photoreceptive field	Small	Small	Large
Photoreceptor response to light	Hyperpolarization (graded potentials)		Depolarization (action potentials)
Signaling single-photon absorption	Yes	No	Yes
Major direct projections	dLGN, SC	dLGN, SC	SCN, OPN, VLPO, LH, DMH, VTA, sPVZ, lHB, mAMY, dLGN, PrGC, SC
Physiological role of light response	Sight (conscious visual perception)		Circadian entrainment, PLR, acute melatonin suppression, sleep-wake cycle regulation, termoregulation, heart-rate regulation, rudimental visual awareness, mood, cognitive performance, alertness
Light signaling at birth	No	No	Yes

*IpRGCs, intrinsically photosensitive retinal ganglion cells; SCN, suprachiasmatic nucleus in the anterior hypothalamus; OPN, olivary pretectal nucleus in the dorsal midbrain; VLPO, ventrolateral preoptic nucleus in the anterior hypothalamus; LH, lateral hypothalamus; DMH, dorso-medial nucleus of the hypothalamus; VTA, ventral tegmental area; sPVZ, hypothalamic subparaventricular zone; dLGN, dorsal lateral geniculate nucleus of the thalamus; PrGC, pregeniculate nucleus (the intergeniculate division of LGN); SC, superior colliculus; lHB, lateral habenula; mAMY, medial amygdala; PLR, pupillary light reflex. References in the article*.

Melanopsin (OPN4) is more ancient in terms of evolution than rod/cone opsins (Provencio et al., 2000) and engages different phototransduction cascades, resulting in cellular depolarization (for review, see Peirson and Foster, 2006). Thus, ipRGCs are very unusual photoreceptors; they are spiking neurons that depolarize in response to light, unlike rods and cones that are highly specialized neurons that respond to light with graded hyperpolarization (for review, see Genovese et al., 2021). Unlike rods and cones, which signal to the brain *via* second- and third-order retinal neurons, ipRGCs communicate light information to the brain directly, i.e. monosynaptically (Berson et al., 2002; Hattar et al., 2002).

Furthermore, ipRGCs differ from other RGCs in many aspects. Unlike canonical RGCs that only show extrinsic (i.e., rod/cone-mediated) responses to light, ‘intrinsically' photosensitive RGCs are called this way because they are capable of responding to light even in the absence of any synaptic input from other retinal neurons (Berson et al., 2002; Hattar et al., 2002). Unlike standard RGCs that project primarily to the visual centers, such as dLGN and SC, ipRGCs form a monosynaptic pathway, namely, the retinohypothalamic tract (RHT), which projects extensively to the whole brain, reaching dozens of brain targets (Berson et al., 2002; Hattar et al., 2002, 2006; Hannibal et al., 2014; Fernandez et al., 2016). Moreover, in addition to using glutamate as their primary neurotransmitter, unlike any other RGCs, ipRGCs also express PACAP (pituitary adenylate cyclase-activating polypeptide), a neuropeptide that is a marker for RHT in mammals (Hannibal et al., 2002, 2004).

**Figure 2:**
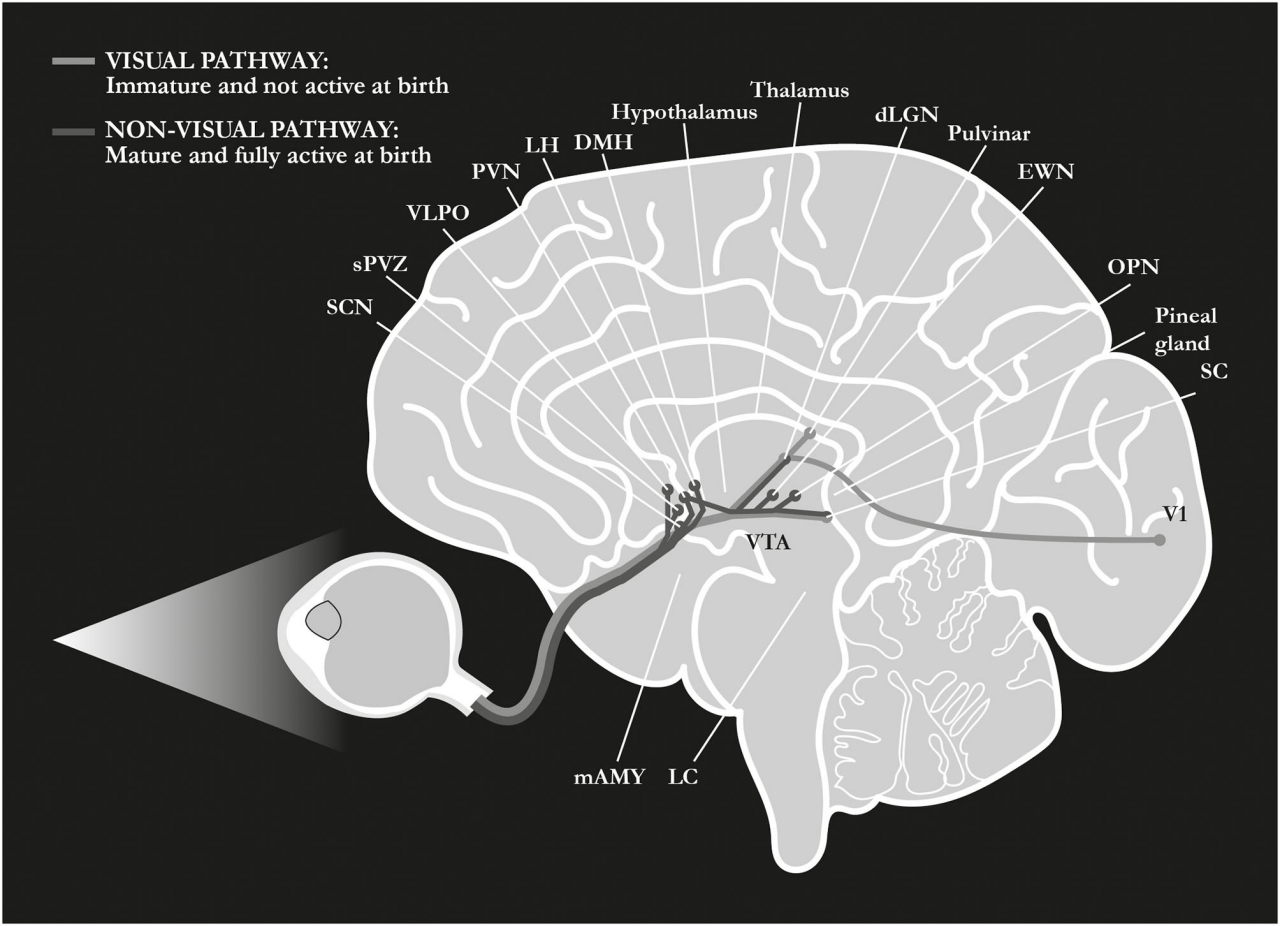
Retinal pathways at birth. At birth, differently from the visual pathway, the non-visual pathway is mature and fully active, allowing for the transferal of the light signal from ipRGCs to many different centers of the brain. SCN, suprachiasmatic nucleus in the anterior hypothalamus; sPVZ, hypothalamic subparaventricular zone; VLPO, ventrolateral preoptic nucleus in the anterior hypothalamus; PVN, paraventricular nucleus of the hypothalamus; LH, lateral hypothalamus; DMH, dorso-medial nucleus of the hypothalamus; dLGN, dorso-lateral geniculate nucleus of the thalamus; EWN, Edinger-Westphal nucleus; OPN, olivary pretectal nucleus in the dorsal midbrain; SC, superior colliculus; V1, primary visual cortex; VTA, ventral tegmental area; LC, locus coeruleus; mAMY, medial amygdala. References in the article.

A major target of the RHT pathway is the suprachiasmatic nucleus (SCN) of the hypothalamus (Berson et al., 2002; Hattar et al., 2002; Hannibal et al., 2004), the master circadian clock that mediates the photic entrainment of the circadian system, thereby regulating almost every aspect of our physiology (Maywood et al., 2006). Other major targets are the olivary pretectal nucleus (OPN), a key center in the control of the pupillary light reflex (PLR), and the ventrolateral preoptic (VLPO) area, a well-established center for the regulation of the sleep-wake cycles. In addition, ipRGCs project directly to further regions of the forebrain and the midbrain, and to the limbic area such as the amygdala and the habenular complex (Hattar et al., 2006; Hannibal et al., 2014; Fernandez et al., 2016; Figure 2). In all mammals, and even more so in primates, ipRGCs also project to visual structures such as the dLGN and the SC (Dacey et al., 2005; Hattar et al., 2006; Hannibal et al., 2014; Liao et al., 2016), and, in humans, they are associated with rudimental light awareness that is retained in totally blind subjects (Zaidi et al., 2007; Vandewalle et al., 2013, 2018; Table 1). Interestingly enough, one single ipRGC has been shown to send input up to five different brain regions and, in some cases, unlike conventional RGCs, to innervate the target bilaterally (Hannibal et al., 2014; Fernandez et al., 2016).

Mapping ipRGC projections has been the first step to discover ipRGCs' multiple functions and, although all their brain targets and functions have been first established in mice (Hattar et al., 2002, 2006; Schmidt et al., 2011; Fernandez et al., 2016; Aranda and Schmidt, 2021), they have now been confirmed in non-human primate and human studies (Dai et al., 1998; Provencio et al., 2000; Hannibal et al., 2004, 2014, 2017; Dacey et al., 2005; Zaidi et al., 2007; Vandewalle et al., 2013, 2018; Daneault et al., 2016; Liao et al., 2016; Prayag et al., 2019a; Mure, 2021).

IpRGCs' role extends far beyond their direct projections, as they also indirectly regulate many physiological processes. In humans, a striking example of this activity is the acute light suppression of the pineal hormone melatonin (Czeisler et al., 1995; Cajochen et al., 2005; Prayag et al., 2019a), and ipRGCs' impact on thermoregulation, the heart rate, mood, alertness and cognitive performance (Cajochen et al., 2005; Zaidi et al., 2007; Vandewalle et al., 2013; Prayag et al., 2019b; Table 1). In particular, unlike nocturnal rodents (mice and rats), for which acute light-induced sleep has been reported (Lupi et al., 2008), fRMI human studies have shown acute alertness caused by blue light *via* the activation of the brainstem, in particular, of the *locus coeruleus* (Vandewalle et al., 2007).

The extremely broad array of ipRGCs' projections and physiological role in response to light are even more relevant if we consider that, in all mammals, ipRGCs only represent a tiny portion of RGCs. In the human retina, where the number of standard visual photoreceptors amounts to ~120 million rods and 6 million cones, ipRGCs account for <1% of millions of RGCs, meaning that there are only a few thousands ipRGCs in the retina (La Morgia et al., 2010; Hannibal et al., 2017; Table 1). Nonetheless, the response to light of these few melanopsin-expressing spiking-neurons, is essential for humans' health and wellbeing and is independent of sight, given that they are retained in blind people lacking functional rods and cones (Czeisler et al., 1995; Zaidi et al., 2007; Vandewalle et al., 2013, 2018).

Another interesting element is that, despite there are very few ipRGCs, they are not homogeneous as a population, rather, they show a remarkable heterogeneity with multiple morphological and functional subtypes identified in all the mammalian species that have been investigated (Tu et al., 2005; Schmidt et al., 2011; Zhao et al., 2014; Hannibal et al., 2017; Mure et al., 2019; Caval-Holme et al., 2022). IpRGCs' extreme variability has been recently confirmed with the discovery of different phototransduction cascades, used by melanopsin in different types of cells (Sonoda et al., 2018), including one single subtype (Emanuel et al., 2017), to drive extreme and flexible diversification of cellular functions and behaviors. In these studies, performed on animal models, melanopsin phototransduction has been shown to be active across a broad range of physiological light intensities including at dim, scotopic light intensities, where only rods were first thought to influence RGC responses. These studies thus indicate that ipRGC-intrinsic melanopsin-mediated phototransduction can be much more sensitive than what previously thought (for review, see Aranda and Schmidt, 2021).

## IPRGCS' Developmental Role at Birth

Animal studies have shown that ipRGCs' impressive functional variability can also be developmental (Lucas and Schmidt, 2019). Some ipRGC types have been found to express much higher melanopsin levels and, consequently, to elicit much more efficient intrinsic light responses at birth and during early postnatal development than in adulthood (Sekaran et al., 2005; Tu et al., 2005; Sexton et al., 2015). While these data suggest different ipRGC functions and behaviors at different developmental stages, the role of such a greater ipRGC light response at birth has not been fully investigated yet.

Although it has still not been entirely determined, ipRGCs' role at birth may be remarkable as ipRGCs are the only active retinal photoreceptors at this stage. Indeed, mammals are functionally blind at birth because rod- and cone-mediated pathways are not fully mature and cannot signal light (Sernagor et al., 2001; Hannibal and Fahrenkrug, 2004; Daum et al., 2017; Bonezzi et al., 2018). However, at birth, ipRGCs respond to light, providing the earliest light-driven signals to the brain (Hannibal and Fahrenkrug, 2004; Sekaran et al., 2005; Sernagor, 2005; Tu et al., 2005; Brooks and Canal, 2013; Sexton et al., 2015; Table 1, Figure 2).

Prior to the maturation of the retinal circuits that mediate visual perception, occurring from a few days to a few weeks after birth, depending on the species (Sernagor et al., 2001; Hannibal and Fahrenkrug, 2004; Hansen and Fulton, 2005; Fulton et al., 2009; Bonezzi et al., 2018), mammalian newborns show several light-induced, ipRGC-mediated functions and behavior, including the pupillary light reflex (PLR; McNeill et al., 2011). At birth, ipRGCs are capable of transmitting light information to the circadian clock, as shown by increased expression of c-*fos*, a marker of neural activity, in the suprachiasmatic nucleus (SCN; Hannibal and Fahrenkrug, 2004; Sekaran et al., 2005). The mammalian circadian system has been shown to be light-responsive as early as birth, in both neonatal rodents, which are born with fused eyelids (Brooks and Canal, 2013), and primate infants at very premature stages (Rivkees, 2007); and ipRGCs' response to light has been shown to start regulating circadian system development as from birth (Rivkees, 2007; Brooks and Canal, 2013; Chew et al., 2017). Moreover, ipRGCs have been shown to have many regulating roles in retinal development (Renna et al., 2011; Kirkby and Feller, 2013; Rao et al., 2013; Tufford et al., 2018), through both a light-dependent activity and a non-light-dependent activity (Chew et al., 2017). For instance, in rodent animal models, where early postnatal spontaneous activity (‘retinal waves') coexists with a light-driven activity until eye opening at postnatal day 12, some subsets of ipRGCs have been shown to regulate these spontaneous waves (Renna et al., 2011; Kirkby and Feller, 2013) and to influence the development of the visual system (Chew et al., 2017; Tiriac et al., 2018).

At birth and during early postnatal development, prior to the maturation of rod and cone pathways, rodent and primate ipRGCs have been shown to form extensive gap-junction networks with other retinal neurons, including other ipRGCs and conventional RGCs (Sekaran et al., 2005; Arroyo et al., 2016; Liao et al., 2016; Caval-Holme et al., 2019). Through this interconnected network, ipRGCs increase amplitude and extention of their own responses to light, simultaneously providing light-sensitivity to conventional RGCs (Caval-Holme et al., 2019). These results, together with the other findings above, are strongly suggestive of the fact that, at birth, the mammalian retina is much more sensitive to light than what previously thought.

In humans, the retinohypothalamic tract (RHT) has been identified at 36 weeks of gestation (GW). In addition, results, which have been extrapolated from a primate model (baboon), show that RHT may be functional from 24 GW onward. These results also show that the fetal SCN oscillates prenatally, in the last trimester of pregnancy (for review, see Rivkees, 2007). In the uterus, the fetal SCN does not have access to the external environment's light-dark cycles, and oscillations that occur in the fetal SCN are due to maternal signals which are transmitted to the fetus during circadian periods (Watanabe et al., 2013; Reiter et al., 2014; Escobar et al., 2021). These signals are considered to be non-photic temporal stimuli for the fetus and include nutritional and hormonal signals, such as melatonin crossing the placenta. Thus, during gestation, the fetus functions as another peripheral oscillator inside the mother (Watanabe et al., 2013; Reiter et al., 2014; Escobar et al., 2021). Maternal circadian signals during gestation are crucial for the development of the fetus and of its circadian system in particular. In preterm births newborns lose circadian information from the mother prematurely, and the development of their circadian rhythms may be impaired by environmental disturbances such as exposition to continuous light in Neonatal Intensive Care Units (NICUs; Watanabe et al., 2013; Hazelhoff et al., 2021). Clinical data show how introducing ‘robust light-dark cycles' in NICUs improves premature newborns' outcomes in terms of increased night-time sleep duration, weight gain, and shorter hospitalization (Watanabe et al., 2013; Hazelhoff et al., 2021). Despite the fact that the biological clock of both full-term and preterm infants (at least at 24 GW) has been found to respond to light as from birth (Hao and Rivkees, 1999), further maturation of the human SCN takes place after birth, in particular, in terms of progressive photic regulation of the circadian system outputs. 24 h sleep-wake rhythms can only emerge between 7 and 16 weeks after term birth, and day-night differences in melatonin production can only be observed as from 3 months of age (for review, see Hazelhoff et al., 2021).

Observational clinical studies have also shown that full-term newborns and preterm newborns, from 30 GW onward, respond to light based on measurements of their pupillary light reflex (PLR) (Robinson and Fielder, 1990). At these early neonatal stages, PLR appears to be exclusively ipRGC-mediated (Hanita et al., 2009; Watanabe et al., 2013; Ikeda et al., 2015), which is supported by electrophysiological studies showing how light response from rods and cones does not begin until a few days or weeks after birth (Hansen and Fulton, 2005; Fulton et al., 2009; Watanabe et al., 2013). Moreover, despite rod/cone pathways not being mature (Hansen and Fulton, 2005; Fulton et al., 2009; Watanabe et al., 2013) and the fovea cones not being fully developed at birth (Abramov et al., 1982), it is well-documented that the newborn has visual behaviors soon after birth (Atkinson, 1984). This observation, coupled with data on the neonatal development of the flash-visual evoked potential (Kraemer et al., 1999; Benavente et al., 2005), suggests that visual behavior may be mediated by the ipRGC pathway in very first few weeks of life.

All these data show that, in all mammals, at birth, the retina is light-responsive *via* the ipRGCs pathway. Data also suggest that, in human newborns (at preterm and at term), this retinal pathway is formed, thus, it is capable of conveying light signals to the brain with a very broad range of projections. Hence, in humans, in response to light at birth, ipRGCs can determine extraordinary fast signaling to the whole brain.

## The Newborn's Likelihood to Survive and the Activation of Specific Neurotransmission Pathways at Birth

The subplate (SP) zone is a highly dynamic and transient cytoarchitectonic compartment of the fetal telencephalic wall (Kostović and Rakic, 1990) made up of a population of glutamatergic and GABAergic neurons, located below the cortical plate (‘sub-plate', SP), in the developing cerebral cortex (for review, see Luhmann et al., 2018). The SP is known to be a phylogenetic novelty that initiates in mammals and develops in primates specifically, and even more so in humans, providing unique transient fetal features and contributing to the introduction of species-specific cell types (Kanold, 2009; Kostović and Judaš, 2010; Miller et al., 2014; Molnár et al., 2020). The SP is an important site of spontaneous endogenous activity which is involved in the development and plasticity of the cerebral cortex. When it was first discovered, it was considered to be the ‘waiting' compartment and the pattern for growing cortical afferents. Today, the SP is known to be an essential element in early cortical functioning and development, and the main site of neuronal differentiation and synaptogenesis. SP acts as an amplifier of early network activity (Kostović and Rakic, 1990; Kanold, 2009; Judaš et al., 2013; Luhmann et al., 2018) and is extensively involved in the formation of cortical columnar structure, the maturation of intracortical inhibition, and the occurrence of ocular dominance plasticity (Hoerder-Suabedissen and Molnár, 2015; Luhmann et al., 2018).

The human SP has been shown to develop the largest compartment of the fetal neocortical anlage, reaching its full functional development at around 24 GW. Initially, retino-thalamocortical connections are specifically guided by the SP and develop in the same period (Hevner, 2000; Kostović and Jovanov-Milosević, 2006; Kostović et al., 2021). [The functional maturation of the suprachiasmatic nucleus (SCN) and the retinohypothalamic trait (RHT) starting from 24 GW have been mentioned in the previous paragraph]. This functional maturation corresponds to well-known clinical evidence on fetal viability, meaning the possibility to live a ‘meaningful life' as from this timepoint (Gatti et al., 2012). We might assume that a neurodevelopmental threshold has been reached at this point so that, at birth, the newborn can react to the extrauterine environment stimuli and thus survive.

Furthermore, as well as other developing circuits, including the retina, the SP gives rise to endogenously generated bursts of neuronal activity, i.e., spontaneous activity transients (SATs). SATs correspond to the intermittent delta-frequency activity observed in conventional EEG recordings, in both fetuses and preterm newborns (Vanhatalo et al., 2005; Vanhatalo and Kaila, 2006). These are essential for developing circuits maturation and the wiring of developing pathways. SATs are found in extremely preterm infants from ~24 GW (Vanhatalo et al., 2005) in EEG analysis. As mentioned before, like other spontaneous patterns of fetal activity, SATs are robust and can endure perturbation, which allows for their activity to be maintained in case of pharmacologic or genetic disruptions in critical circuit development at the fetal stage. SATs recover quickly after perturbation to ensure proper circuit development (Blankenship and Feller, 2010).

In the first few days after birth, they are associated with regular, sensory-driven waves, which correlate with response to the external environment's stimuli. Then, their frequency progressively diminishes until they can no longer be detected on EEG (Kanold, 2009; Kostović et al., 2021). In addition, delta-brush is the dominant pattern of rapid oscillatory activity (8–25 Hz) in the human cortex during the third trimester of gestation. Like SATs, it disappears during the perinatal period. In particular, this pattern disappears around the end of the first postnatal week. Spontaneous and evoked delta-brushes can be observed in the somatosensory cortex of premature human neonates of 28–32 weeks of postconceptional age (Milh et al., 2007; Luhmann et al., 2014, 2018, 2022; Molnár et al., 2020; Shibata and Otsubo, 2020; Kidokoro, 2021). Delta brushes in preterm human infants correlate with the so-called spindle bursts, recorded in rodents during the early postnatal period (Colonnese et al., 2010; Colonnese and Khazipov, 2012; Yang et al., 2016). These features could lead to a physiologically different EEG profile between the fetus, the preterm newborn, and the full-term newborn. In particular, endogenous bursts of spontaneous neuronal network activity, such as SATs, are linked to a trophic and developmental function. On the contrary, the EEG profile in newborns is characterized by patterns of brain activity that are mainly associated with an extrauterine stimulus-dependent function.

The SP neurons' spontaneous activity is critical for the maturation of GABAergic circuits. In the fetal period, GABA and glycine have an excitatory activity, in contrast with their postnatal inhibitory function (Ben-Ari et al., 2007). The early GABA excitatory function has a developmental role in neuronal differentiation and circuit formation (Ben-Ari et al., 1989, 2007, 2012; Cherubini and Ben-Ari, 2011; Ben-Ari, 2014). The switch in GABA function might be linked to the SP activity and seems to be connected to sensory input (Peerboom and Wierenga, 2021). For instance, in animal models, an excitatory function has been reported in SP GABAergic neurons and the role of SP neurons in the maturation of neocortical GABAergic inhibition has been described (Kanold, 2009; Kanold and Luhmann, 2010; Luhmann et al., 2014, 2018). When the time of birth is approaching, a progressive switch in GABA activity, from excitatory to inhibitory, begins due to the up-regulation of potassium-chloride co-transporter (KCC2) and gradual decrease in intracellular chloride concentration, which is evident during the third trimester of gestation (Vanhatalo et al., 2005).

Moreover, in animal models, oxytocin has been shown to play a relevant role in the switch of GABA function, during delivery. Oxytocin is an antistress and a neuroprotective agent, working through abrupt reduction in intracellular chloride levels, which are high *in utero*. This mechanism strenghtens GABAergic inhibition and modulates the generation of the first synchronized patterns of cortical networks (Khazipov et al., 2008; Feldman et al., 2016; Ben-Ari, 2018). The progressive GABA switch results in an inhibitory activity at birth within the context of a neural resetting that takes place at this stage (Ben-Ari et al., 2007; Sedmak et al., 2016). This is compatible with the newborn's need to cope with and react to extrauterine stimuli (Gatti et al., 2012). A neuroprotective function has also been attributed to GABA inhibitory activity at birth (Ben-Ari et al., 2007). This protective role may not be necessary in the intrauterine environment, where the fetus lies in a condition of homeostasis (Lagercrantz and Slotkin, 1986).

Moreover, GABA switch can be induced by light stimulation of the retina at birth; at the same time, GABA activity may determine a developmental change in retinal waves' dynamics (Sernagor et al., 2003; Maccione et al., 2014; Peerboom and Wierenga, 2021). This suggests how GABA protective function in the passage from the fetal to the neonatal stage may protect the newborn from extrauterine stimuli, e.g., intense retinal stimulation by light. Reciprocal modulation between GABA and RGCs-mediated retinal activity (Maccione et al., 2014) may have implications for the biological dynamics that occur at birth in response to light.

Although GABA functional switch from a prenatal to a neonatal condition is a slow and gradual process that does not occur suddenly at birth, it is worth noticing how GABA temporarily to inhibition at approximately the time of labor, following release of maternal oxytocin; then, it resumes its excitatory function and then switches gradually back to inhibition, in animal studies (Tyzio et al., 2006; Ben-Ari, 2018). Birth is associated with a dramatic, abrupt, oxytocin-mediated, short-lasting reduction in chloride to levels that are not observed before nor later (Tyzio et al., 2014).

This temporary GABA switch may play a protective function against brain trauma and glutamate toxicity (due to hyperexcitability) linked to labor and birth. These ‘back and forth' functional changes indicate the presence of a sudden rearrangement of neurotransmitters' patterns at birth. GABA can play a fundamental role in the modulation of the brain response to bombardment by external stimuli at birth, including stimulations of the retina.

Alterations in the polarity of GABA activity have been extensively investigated, as GABA is developmentally regulated and highly susceptible to insults that restore immature excitatory actions. Several crucial events begin before birth, such as the oxytocin-mediated GABA shift. In experimental models, intrinsic alterations in activity and morphology have been shown to occur during delivery (Bonnet-Brilhault et al., 2018; Cloarec et al., 2019; Caly et al., 2021). The lack of GABA shift has been found in neurodevelopmental disorders, such as autism spectrum disorder (ASD), Rett Syndrome, and other disorders, which may be explained by its regulating and neuroprotective role (He et al., 2014; Tyzio et al., 2014; Ben-Ari, 2015; Lozovaya et al., 2019; Ben-Ari and Cherubini, 2022). One single administration of bumetanide can restore the correct polarity inhibitory action of GABA, however, it may impair behavior in the long term, once pups become adults (Tyzio et al., 2014). This pleads for the crucial relevance of the events occurring at birth. Even when respiratory difficulties have been reported in experimental models (Lozovaya et al., 2019), the GABA shift blockage does not prevent birth, the first breath and survival in the presence of these neurodevelopmental disorders, suggesting that other mechanisms, together with GABA polarity shift, are part of the process of birth.

The neonatal brain is the result of complex and genetically programmed trajectories. The prenatal dysregulation of these mechanisms can lead to the onset of disorders; however, prompt intervention at birth, or immediately after birth, can represent a new strategy of intervention, going as far as minimizing impaired prenatal brain programming (Ben-Ari and Spitzer, 2010; Tyzio et al., 2014; Thomason et al., 2021). For these reasons too, it is crucial to discover the mechanisms occurring at birth, when the CNS comes into contact with an extraordinarily new environment. The purpose of this study is to identify a new neurodevelopmental event that begins at birth as a reaction to the intense, irreversible, and new light stimulation from the extrauterine environment.

Furthermore, a specific set of light-induced Immediate Early Genes (IEGs), functioning as neuronal markers, have been described in studies on experimental models in mammals. In particular, a dramatic photo-dependent induction of IEG *c-fos* has been observed at birth (Hannibal and Fahrenkrug, 2004; Sekaran et al., 2005; Matěju et al., 2009; Brooks and Canal, 2013). A functional change in *c-fos* induction as a reaction to light has been reported at birth (Weaver and Reppert, 1995). It is intriguing to notice that IEGs have a pivotal role in the plastic changes of the CNS in terms of sensory perception, including vision, and that their activation happens within minutes from stimulation, as early as birth (Pinaud, 2004; Pinaud et al., 2006; Franceschini et al., 2020). In conclusion, as possible biomarkers for human life, IEGs could be considered to be putative markers for the activation of the brain in response to extrauterine environment stimuli, to light in particular.

## Discussion

In humans, studies on brain functions are not easy to be performed as they can be invasive and unfeasible, particularly so on pregnant women, fetuses, and newborns. On the other hand, human neurodevelopment has unique and specific features that are associated with extraordinary mental capabilities, including cognitive and affective abilities, which distinguish humans from other primates; hence, they cannot be inferred from animal model studies (Bibb et al., 2001; Clancy et al., 2001; Clowry et al., 2010; Judaš et al., 2013; Foster et al., 2020; Luhmann et al., 2022). Herein, human studies have been referred to when available, and animal studies have been considered taking into account all relevant differences.

Birth is a unique event in life, and much still remains to be discovered about it. Few researchers only have so far taken into account the impact of the extrauterine environment, with its intense stimuli, on birth (Schwindt et al., 2018; De Asis-Cruz et al., 2020; Schmidt Mellado et al., 2022). The GABA excitatory/inhibitory shift may suggest a complete neurofunctional change from the fetal to the neonatal status (Long et al., 2005; Ben-Ari et al., 2007; Gatti et al., 2012; Sedmak et al., 2016). The onset of an inhibitory activity can be associated with the newborn's response to the bombardment by extrauterine stimuli, different from intrauterine homeostasis (Maccari et al., 2017; André et al., 2018; Schwindt et al., 2018; Fagioli, 2019; De Asis-Cruz et al., 2020; Schmidt Mellado et al., 2022; Figure 2). In particular, GABA switch can be induced by light stimulation of the retina at birth; at the same time, GABA activity may determine a developmental change in retinal waves' dynamics (Sernagor et al., 2003; Maccione et al., 2014; Peerboom and Wierenga, 2021).

The impact of the new environment should be considered in order to understand what happens to the brain at birth, in particular during the time of ~20 s before the first breath occurs (Polese et al., 2021). This ‘hypothesis and theory' article identifies light as a potential stimulus for the activation of the CNS at human birth *via* the melanopsin-dependent retinal non-visual pathway. Light may be considered to be the main trigger of a sudden shift of the brain from a prenatal pattern of functions to a neonatal setup, thereby activating the first breath. Light is the most extensively available source of energy the mammalian brain can catch and process, as it is always available in the environment, in darkness too. It has the properties that are needed from a stimulus involved in at-birth dynamics; in particular, light acts fast, in only a few seconds, between the exit from the uterus and the newborn's first breath and crying. Light photons are the only components in the extrauterine environment that have the following features: novelty, availability, constancy, speed, and power. Moreover, light is energy, meaning it does not have mass; hence, it can cross the pupils reaching the brain *via* the retina.

**Figure 3:**
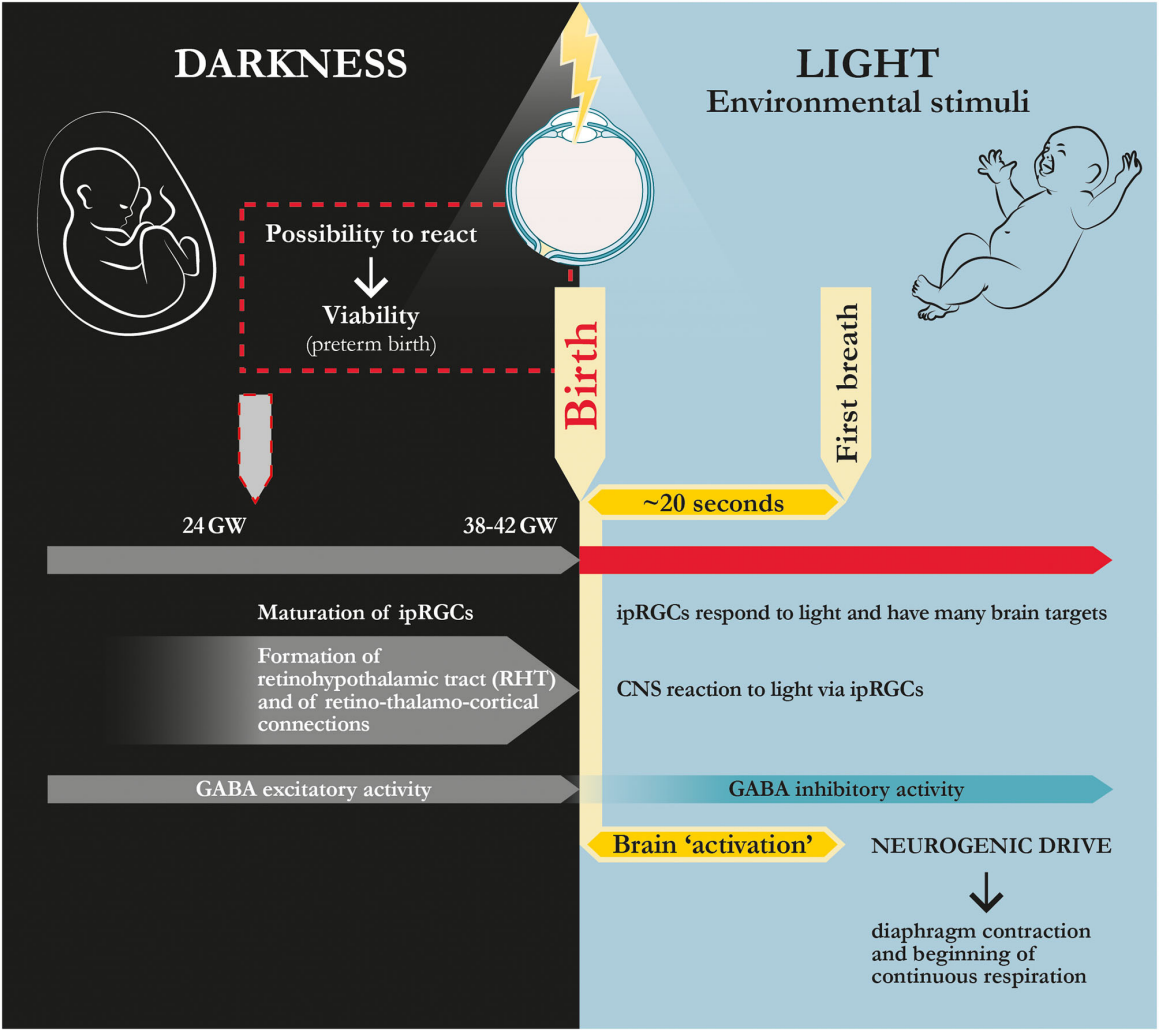
Physiological dynamics at human birth: ‘coming to light.' In humans, the fetus becomes viable at ~24th gestational week when retinohypothalamic tract (RHT) and retino-thalamo-cortical connections are formed (Hevner, 2000; Gatti et al., 2012; Fagioli, 2019). According to this hypothesis and theory article, maturation of these pathways allows for the transferal of the signal induced by light/photon stimulus from retinal active photoreceptors (ipRGCs) to the whole brain, thereby rapidly activating respiratory nuclei and driving the first breath. GW, gestational weeks; ipRGCs, intrinsically photosensitive retinal ganglion cells.

The melanopsin-expressing, intrinsically photosensitive retinal ganglion cells (ipRGCs) mainly perform functions that occur beyond conscious perception (‘non-visual'), e.g., they are part of the biological clock that governs the rhythm of life (Table 1). Moreover, unlike the standard visual system, this pathway is mature and fully active at birth, with a more efficient intrinsic response to light than during adulthood (Hannibal and Fahrenkrug, 2004; Sekaran et al., 2005; Sernagor, 2005; Tu et al., 2005; Caval-Holme et al., 2019). Although some of the ipRGCs' neonatal functions have been identified, the role of such a greater response to light at birth has not yet been fully explained. Moreover, unlike rods and cones, ipRGCs project widely throughout the brain (Figure 2, Table 1), and fMRI human studies show a broad activating effect of blue light in the brain, starting from subcortical structures, i.e., the thalamus, the hypothalamus, and the brainstem, specifically the *locus coeruleus* (Vandewalle et al., 2007, 2013; Daneault et al., 2016). Thus, at birth, the light signal may indirectly (polysinaptically) reach the brainstem respiratory nuclei *via* the ipRGCs pathway. In that regard, the Kölliker-Fuse (KF) nucleus, a pontine reflex blocking center, has been shown to reverse its function abruptly at birth, activating the pontine facial/parafacial complex, which, in turn, triggers the medullary pre-Bötzinger nucleus, thereby determining a ‘neurogenic drive' for the contraction of the diaphragm. This network activation might generate ‘the first and strongest respiratory effort', namely, the first breath (Ottaviani, 2014; Lavezzi et al., 2016). Hence, based on our assumption, the first breath follows a light-induced brain reaction (Fagioli, 2019; Figure 3).

Recent studies show how ipRGCs can be active across a broad range of physiological light intensities including in darkness, i.e., at dim, scotopic light intensities, where a lower amount of photons is present (for review, see Aranda and Schmidt, 2021). In addition, it has been clinically observed that, at birth, the newborn presents with a condition of physiological mydriasis, due to the extraordinary amount of catecholamines released following prolonged and intense stress characterizing the birth process. This suggests that the eyes are to be as much anatomically open to light as possible (Lagercrantz and Bistoletti, 1977; Lagercrantz and Slotkin, 1986) in order to catch more photons.

Furthermore, we have considered that exposure to light inside the uterus, including cases of *in-utero* surgery, does not activate the CNS. This can be due to differences in the fetal brain from the neonatal brain (Doria et al., 2010; Viola et al., 2011; Counsell et al., 2019; Kostović et al., 2021). For instance, studies on animal models suggest that ipRGCs have different roles at different developmental timepoints (Lucas and Schmidt, 2019). In particular, they suggest that ipRGCs' functions in the uterus, such as their role in retinal vascular patterning (Rao et al., 2013), can differ from their functions after birth (Rivkees, 2007; Brooks and Canal, 2013; Sexton et al., 2015; Lucas and Schmidt, 2019; Escobar et al., 2021).

It has been observed that mice undergoing knockout of the OPN4 gene, which encodes for melanopsin, remain viable, which suggests the onset of compensatory mechanisms (Rao et al., 2013). For instance, other photosensitive opsins are also expressed in the mammalian CNS (Shiosaka and Ishikawa, 2011). OPN4 knock-out mice develop abnormal retinal vasculature comparable to what has been observed in dark-reared animals. Thus, in mice, ipRGCs' light response *in utero* has been considered to be fundamental for the vascular patterning of the retina (Rao et al., 2013). However, to what extent this applies to human fetal eye development still remains to be investigated (Hazelhoff et al., 2021; Luhmann et al., 2022).

In humans, during the last trimester of gestation, the fetal SCN oscillates due to maternal non-photic stimuli (such as melatonin), which are transmitted to the fetus during circadian periods. Instead, after birth, there is a progressive photic regulation of the circadian system outputs, with marked rhythms in sleep-wake phenomena and hormone secretion. This suggests that the human fetal SCN does not have access to the external environment's light-dark cycles (Watanabe et al., 2013; Reiter et al., 2014; Escobar et al., 2021; Hazelhoff et al., 2021). In fMRI human studies, visual stimuli fail to evoke responses in the V1 visual cortex of an at-term fetus (>36 GW; Fulford et al., 2003). Methodological limitations have to be considered in order to understand this null finding. For instance, it is difficult to know if the stimulus is effectively delivered to the eyes (Anderson and Thomason, 2013). On the other hand, data show that the rapid development of the visual system occurs postnatally (Bengoetxea et al., 2012). Thus, ontogeny of the human visual pathway is likely to be complete at the time of birth, and the lack of sensory-driven experience *in utero* could explain this result by Anderson and Thomason (2013).

Finally, it is worth noticing how neurodevelopmental processes are unbreakable. The spontaneous network activity of developing neural circuits (such as retinal waves, SAT's and delta-brushes) is robust as it is constantly ensured and actively maintained (Blankenship and Feller, 2010; Arroyo and Feller, 2016; Molnár et al., 2020). In animal models, some ipRGC subtypes seem to function in parallel with spontaneous retinal waves in the developing retina (Caval-Holme et al., 2019), and it can be assumed that they modulate wave properties in a light-independent way (Chew et al., 2017). However, a recent study has shown how, at birth, a particular ipRGC subset produces a response to light that is independent from retinal waves, thus providing the capability to distinguish the new light stimulus from developmental patterns of spontaneous activity (Caval-Holme et al., 2022).

This ‘hypothesis and theory' article could pave the way to a paradigm shift on human birth. Should our assumption be confirmed, we could acknowledge the role of the brain reacting, *via* the retina, to light stimulation from the extrauterine environment, and its activating new functions at birth which is essential for survival. It could be an attempt to solve the enigma of the ‘first breath' as the beginning of human life, focusing researchers' attention on the activation of the brain, and, thus, on the beginning of the functioning of the mind as the primal condition for human life.

## Limitations and Future Directions

This article proposes a thesis and a theory that have been based on evidence available so far but which have still not been tested directly. The aim is to stimulate further research on human birth at a multidisciplinary level. So far, no study has specifically focused on the processes that happen when the newborn comes to light, or the characterization of the brain's physiological responses to the extrauterine environment. The lack of scientific knowledge supports the common thinking that human life starts with the first breath, which is not accurate (Polese et al., 2021). Although we cannot hope for an immediate breakthrough, also due to methodological limits, new studies can contribute to informing our research (Luhmann et al., 2022). In particular, research based on resting-state fMRI (Anderson and Thomason, 2013) can open new scenarios on the human brain's early development. Also, functional ultrasound imaging (fUS) has been recently used to assess neonatal brain functions (Demene et al., 2017; Baranger et al., 2021). FUS enables to identify markers of cerebral activity based on intrinsic resting-state functional connectivity for better and more specific characterization of the brain maturation, as early as birth (Baranger et al., 2021). This study is addressed to peers who work in different medical fields, from neurophysiology to neurology and psychiatry, and to researchers, scholars, and professionals of social science, ethics or law.

## Data availability statement

The original contributions presented in the study are included in the article/supplementary material, further inquiries can be directed to the corresponding author.

## Ethics statement

Written informed consent was obtained from the individual(s) for the publication of any identifiable images or data included in this article.

## Author contributions

DP and MLR contributed to the article and its layout to the same extent, searching for literature data, and writing the article. AM, MarF, FF, and PP discussed the background, read, and commented the whole article. MasF who passed away a few years ago, was the first scientist who in his books on Human Birth Theory, conceptualized that human beings' first breath is triggered by the activation of the brain following its reaction to light, formulated the assumption and the relevant theory, agreed on the layout of the article, and supervised all the work done on its first draft. All authors contributed to the article and approved the submitted version.

## Funding

This article was supported by the grant ord. prot. 43/2022 from the Ph.D. funds, Department of Neuroscience, Mental Health and Sensory Organs NESMOS, Sapienza University of Rome, Rome, Italy.

## Conflict of Interest

The authors declare that the research was conducted in the absence of any commercial or financial relationships that could be construed as a potential conflict of interest.

## Publisher's Note

All claims expressed in this article are solely those of the authors and do not necessarily represent those of their affiliated organizations, or those of the publisher, the editors and the reviewers. Any product that may be evaluated in this article, or claim that may be made by its manufacturer, is not guaranteed or endorsed by the publisher.
